# The RH5-CyRPA-Ripr Complex as a Malaria Vaccine Target

**DOI:** 10.1016/j.pt.2020.04.003

**Published:** 2020-06

**Authors:** Robert J. Ragotte, Matthew K. Higgins, Simon J. Draper

**Affiliations:** 1 The Jenner Institute, University of Oxford, Oxford, OX3 7DQ, UK; 2 Department of Biochemistry, University of Oxford, Oxford OX1 3QU, UK

**Keywords:** malaria vaccine, *Plasmodium falciparum*, erythrocyte invasion, RH5, CyRPA, Ripr

## Abstract

Despite ongoing efforts, a highly effective vaccine against *Plasmodium falciparum* remains elusive. Vaccines targeting the pre-erythrocytic stages of the *P. falciparum* life cycle are the most advanced to date, affording moderate levels of efficacy in field trials. However, the discovery that the members of the merozoite PfRH5-PfCyRPA-PfRipr (RCR) complex are capable of inducing strain-transcendent neutralizing antibodies has renewed enthusiasm for the possibility of preventing disease by targeting the parasite during the blood stage of infection. With Phase I/II clinical trials now underway using first-generation vaccines against PfRH5, and more on the horizon for PfCyRPA and PfRipr, this review explores the rationale and future potential of the RCR complex as a *P. falciparum* vaccine target.

## Highlights

The antigens PfRH5, PfCyRPA, and PfRipr can induce strain-transcendent neutralizing antibodies, and all three targets are essential and highly conserved.
PfRH5, PfCyRPA, and PfRipr form a stable complex (RCR) that is involved in the induction of an erythrocytic calcium spike during merozoite invasion.
Passive transfer of anti-CyRPA and anti-PfRH5 antibodies can protect against blood-stage *P. falciparum* in animal models.
Structural studies have mapped out the first known critical inhibitory epitopes on PfRH5 and PfCyRPA which can be used for next-generation vaccine design.
Early results from the first PfRH5 vaccine clinical trials have been reported with more anticipated soon, which will help guide the development of RCR-based vaccines.

## Malaria Vaccine Status

Global malaria mortality and morbidity has declined sharply in recent decades. Still, in 2018 there were 228 million infections resulting in 405 000 deaths, disproportionately shouldered by the developing world [1]. Despite progress, malaria continues to be an intractable global health threat. Improved access to insecticide-impregnated bed nets, vector control, and the availability of effective antimalarial medications have been the cornerstones of global malaria control efforts and will continue to play indispensable roles. However, even optimal deployment of current tools will still leave elimination in high-transmission settings unattainable [2].

A highly effective malaria vaccine would be one way to achieve further reductions in the global malaria burden. The observation that passive immunoglobulin (Ig) transfer confers immunity against malaria suggests that a malaria vaccine is conceivable [3]. One malaria vaccine candidate from GlaxoSmithKline, RTS,S/AS01, targets the circumsporozoite protein (CSP) and has now progressed beyond Phase III and into pilot implementation trials. This is a milestone for the malaria vaccine field and provides an important proof-of-principle for this approach; however, improvement is still required given that RTS,S/AS01 affords only partial protection of modest duration [4]. Nonetheless, this partial success gives strong impetus for continued effort and investment to develop a more effective next-generation malaria vaccine.

Currently, the field awaits proof-of-concept for a substantially improved CSP-based vaccine, while whole-sporozoite strategies still face challenges with regard to scalability, immunogenicity in African infants, and breadth of protection [5,6]. An alternative and complementary approach would be to include a vaccine targeting the subsequent pathogenic blood stage of infection; this would have the potential to protect against malaria death, disease, and transmission.

**Figure 1:**
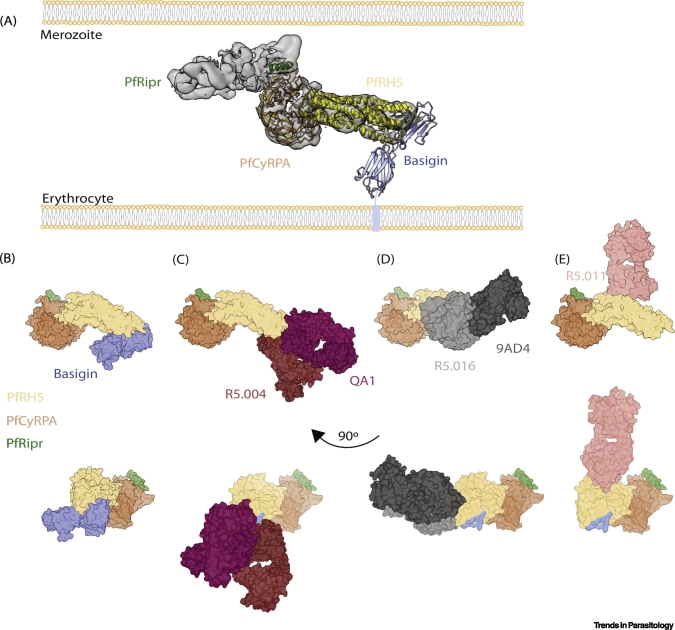
Mapping the Critical PfRH5 Epitopes on the RCR Complex. (A) An illustration of the RCR complex binding basigin on the human erythrocyte based on the cryo-EM structure (EMD-9192, PDB: 6MPV) and crystal structure of PfRH5:basigin (PDB: 4U0Q). (B) RCR bound to basigin. (C) RCR binding basigin-blocking Fabs QA1 (PDB: 4U1G) and R5.004 (PDB: 6RCU). (D) RCR bound to basigin proximal Fabs R5.016 (PDB: 6RCU) and 9AD4 (PDB: 4U0R). (E) RCR bound to synergistic noninhibitory Fab R5.011 (PDB: 6RCV). The basigin binding site on PfRH5 has been coloured blue where basigin is absent in the lower panels. Figure produced using Chimera [87]. Abbreviations: cryo-EM, cryoelectron microscopy; RCR, PfRH5-PfCyRPA-PfRipr; PDB, protein data bank.

This review focuses on the recently described PfRH5-PfCyRPA-PfRipr (RCR) complex, an elongated protein trimer formed on the *P. falciparum* merozoite surface that binds to erythrocyte basigin, as a new and highly promising next-generation **blood-stage vaccine** (see Glossary) candidate (Figure 1A). It outlines the structure and function of each component, the available data on *in vitro* and animal-model efficacy, as well as human immunogenicity. Though there are six species of *Plasmodium* parasite that can cause disease in humans, among these six, RH5 is unique to *P. falciparum,* while orthologues of CyRPA and Ripr are present in all [7]. *P. falciparum* is responsible for the largest portion of global malaria deaths and is the subject of this review. Going forward, any reference to ‘malaria’ will refer to *P. falciparum* malaria, unless otherwise specified.

## Blood-Stage Vaccination

The mainstay approach to blood-stage vaccine development has been to induce antibodies that target the invasive merozoite form of the blood-stage parasite [8]. Unlike a **pre-erythrocytic vaccine**, a blood-stage vaccine would not necessarily need to provide sterile immunity against infection, although this remains the ultimate goal for vaccine development efforts. Rather, an intervention that sustained high-level reductions in blood-stage parasitaemia would still prevent death and clinical episodes of malaria – potentially making an immense contribution to the control of the malaria disease burden. Moreover, rodent and non-human primate models of vaccine-controlled *Plasmodium* blood-stage infection also suggest that residual parasites would ultimately be cleared (likely via the induction of naturally acquired immune responses) [9,10]. The distinction, then, between a successful and unsuccessful vaccine is not categorical based on the presence/absence of infection but will ultimately necessitate monitoring of clinical malaria in field efficacy trials.

Historically, two obstacles that have prevented the development of an effective anti-merozoite blood-stage vaccine were the parasite’s reliance on redundant host–pathogen interactions, which provide alternative erythrocyte invasion pathways if one is blocked, and polymorphism of essential ligands, which results in strain-specific immunity. The latter was seen during field trials of the once-leading blood-stage vaccine target apical membrane antigen 1 (AMA1), which only conferred strain-specific partial efficacy due to its polymorphic nature [11]. However, these challenges appear to have been overcome with the discovery of the highly conserved and essential RCR complex.

Further challenges include the speed of erythrocyte invasion and the parasite’s complex life cycle. The former necessitates a high concentration of antibody, which must act in a short window of opportunity [8,12], while the parasite’s multi-stage, multi-host cell life cycle makes expression of even the most compelling vaccine targets ephemeral, restricted spatially and temporally over the course of infection. Indeed, in all likelihood, the first highly effective malaria vaccine will rely on multiple components targeting different stages of the parasite’s life cycle (Box 1) [8,13., 14., 15.].

Box 1 **Erythrocyte Invasion**

*P. falciparum* has a complex multi-stage life cycle. It begins with injection of sporozoites into the bloodstream during an *Anopheles* mosquito blood meal. The sporozoites migrate to the liver where they invade hepatocytes. Within the liver, the parasites replicate asexually before being released into the bloodstream approximately 7 days later. The blood stage of infection is responsible for the clinical symptoms of malaria. During this stage of infection, the parasites progress from ring-stage trophozoites to schizonts before egress 48 h after initial invasion, releasing merozoites into the blood.

Erythrocyte invasion by *P. falciparum* was recently reviewed in depth [35] and will be summarized only briefly. This highly complex cellular invasion event is significant from the perspective of vaccine development because it is one of the few times during the parasite’s life cycle that it is directly exposed to the immune system, thereby providing a brief window during which clinical illness may be averted.

Host erythrocyte invasion occurs in three steps: initial attachment, tight-junction formation, and invasion. Initial attachment is likely mediated by merozoite surface proteins (MSPs). Following these low-affinity interactions, tight attachment occurs, which is mediated by erythrocyte-binding-like (EBL) and reticulocyte-binding-like homolog (RH) gene families [35]. Subsequently, the merozoite reorients, enabling membrane deformation and the attachment of PfRH5 to the host receptor basigin, which is associated with a calcium spike within the erythrocyte [24,27]. Next comes the formation of the tight junction. Here, the parasite supplies its own receptor by inserting a complex of rhoptry neck proteins (RONs) into the host red cell membrane, which is then bound by AMA1 [35,74., 75., 76.].

Invasion concludes with the propulsion of the tight junction from the apical end of the parasite rearward via an actin-myosin motor [77]. This results in the engulfment of the merozoite within the **parasitophorous vacuole**. This entire process in completed in approximately 30 s, meaning that there is only a narrow window during which invasion can be averted [78]. Once inside the erythrocyte, the merozoite divides via schizogony over the course of 48 h resulting in 16–32 new merozoites per infected cell [79], before egress and release to begin a fresh round of erythrocyte invasion.

Alt-text: Box 1

## *P. falciparum* Reticulocyte-binding Protein Homolog 5 (PfRH5)

### Structure and Function

PfRH5 is currently the most advanced next-generation blood-stage vaccine candidate antigen. It was identified as a member of the PfRH family both through a homology-based search [16], and through linkage analysis [17]. PfRH5 is a 63 kDa protein expressed during the mature schizont stages, localized to the **rhoptries** [18,19]. It is processed and cleaved to a 45 kDa form, which is subsequently shed into parasite culture supernatant [18,19]. The structure of PfRH5 reveals a kite-like architecture formed from the coming together of two three-helical bundles [20,21]. This same fold was seen in the *P. vivax* reticulocyte-binding protein 2a (RBP2a) invasion ligand, demonstrating the potential for versatile binding properties [22].

PfRH5 lacks a transmembrane domain or **glycosylphosphatidylinositol (GPI) anchor**. Initially, cysteine-rich protective antigen (PfCyRPA) was thought to anchor it to the merozoite surface; however, it has since been shown that PfCyRPA also lacks a GPI anchor [23., 24., 25.]. P113, a GPI-anchored protein, was thereafter shown to bind the PfRH5 N terminus, suggesting a possible role in invasion [25]. This is complicated by the observation that P113 is also known to associate with the PTEX translocon, meaning that any role alongside the RCR surface complex would be an additional, seemingly unrelated, biological function [26]. Further investigation is needed to understand the role of P113 in erythrocyte invasion and whether it can be targeted by neutralizing antibodies.

PfRH5 is indispensable for human erythrocyte invasion [27]. As such, it is refractory to genetic deletion [17,19]. Intriguingly, PfRH5 orthologues are found in other species belonging to the *Lavarenia* subgenus, which includes parasites that infect chimpanzees and gorillas, but they are not found in the other species of *Plasmodium* that infect humans, indicating a unique role in *P. falciparum* invasion of human erythrocytes [7,17,28]. Genomic studies identified the transfer of an 8 kb stretch of DNA encoding both PfRH5 and PfCyRPA from the gorilla parasite *P. alderi* to an ancestor of *P. falciparum* as a critical event in the emergence of *P. falciparum* as a lethal human parasite [29,30], while polymorphisms in *PfRH5* play a critical role in defining species-specificity of host cell receptor binding, thereby determining infectivity [31., 32., 33., 34.].

In humans, PfRH5 binding to basigin plays an essential role in invasion, acting downstream of membrane deformation (Box 1) [24,27,35]. Binding of PfRH5 to basigin is required for the induction of a spike in calcium within the erythrocyte, which is blocked when merozoites attempt to invade in the presence of anti-PfRH5, anti-PfRipr, or anti-basigin antibodies or soluble basigin [24]. This calcium spike is correlated with echinocytosis, suggesting that it may be important for invasion [35]. However, there are conflicting data on whether recombinant PfRH5 on its own or in complex is able to induce elevated intracellular calcium concentrations within the erythrocyte [24,36].

One model of invasion suggests that, upon binding to erythrocyte basigin, PfRH5 inserts into the membrane, alongside PfRipr, where it unfolds, potentially forming a pore [37]. However, there is little evidence for this as the structural and biochemical properties of PfRH5 are not consistent with membrane insertion or unfolding; structures of PfRH5 in complex with a variety of antibodies and erythrocyte basigin, in a variety of crystallization conditions, are essentially identical, suggesting a robust and stable protein fold [20,21,38].

### Antibody Response to PfRH5

In malaria, cross-strain neutralizing vaccine targets were thought to be subject to considerable natural immune pressure; thus, typically, such targets showed wide antigenic diversity. However, PfRH5 appears to be the first identified exception to this dogma [39]. Indeed, vaccine-induced antibodies that mediate parasite neutralization *in vitro* were effective across all *PfRH5* SNPs tested, including the five most common (Table 1) [38,40., 41., 42.].

**Table 1:** Single-nucleotide Polymorphisms in the RCR Complex with Minor Allele Frequency >5%

Protein	Mutation	Minor allele frequency (%)	Refs
PfRH5	H148D	18.41	[86]
	Y147H	17.39	
	S197Y	16.36	
	C203Y	13.50	
	I410M	9.40	
PfRipr	Y985N	39.67	
	V190A	13.11	
	I1039M	10.83	
	A755G	9.13	
	H511R	7.97	
	L673V	6.93	
PfCyRPA	R339S	14.84	

In light of these vaccine data, interest arose in naturally acquired anti-PfRH5 responses. Following natural infection, as opposed to vaccination, anti-PfRH5 seropositivity was associated with reduced parasitaemia in a cohort of children from Papua New Guinea, while a study in Malian children drew an association between anti-PfRH5 seropositivity and protection from febrile malaria [43., 44., 45.]. Although there appears to be an association between naturally acquired anti-PfRH5 antibodies and protection from clinical *P. falciparum* malaria, PfRH5 also appears to lack the inherent immunogenicity observed with many other well described *P. falciparum* merozoite proteins in the context of natural exposure. Indeed, when antibody responses in previously infected Kenyan adults were examined, researchers found markedly reduced antibody levels to PfRH5 compared to PfAMA1 and nine other blood-stage vaccine targets [39]. This immunoepidemiological observation has now been consistently observed across numerous studies, with a number including large panels or arrays of malaria antigens [43,46., 47., 48.].

The reason(s) why PfRH5 is poorly immunogenic in natural malaria infection remain unclear, although these probably relate to low antigen abundance, with PfRH5 protein lost in a sea of many other highly abundant immunodominant (decoy?) targets, such as MSP1. Early concerns about issues of human tolerance to PfRH5 due to self-similarity have floundered in light of data confirming strong vaccine-induced immune responses against PfRH5 following small animal, *Aotus* monkey, and now human vaccination [9,39,42].

At present, anti-PfRH5 **monoclonal antibodies** (**mAbs**) are the most potent among those targeting each of the three antigens within the RCR complex (Table 2), as assessed by the standardized *in vitro* **growth-inhibition activity** (**GIA**) **assay** (Box 2). Notably, this assay was shown to strongly associate with protection outcome in vaccinated *Aotus* monkeys and was then confirmed as a mechanistic correlate (i.e., causative of protection) following mAb **passive transfer** [9,49], with similar results also obtained in humanized mice [50]. The most potent reported anti-PfRH5 mAb, R5.016, has a one-cycle GIA **EC**_**50**_ at 9.6 μg/ml, matching or surpassing many of the anti-PfRH5 polyclonal sera that have been tested (Table 2) [38,40,41,51,52].

**Table 2:** Neutralizing Monoclonal Antibodies to the RCR Complex

Antibody	Target	Isotype	Species	Concentration (mg/ml)	GIA (%)^a^	Cross-strain neutralizing	Binding details	Refs
BS1.2	PfRH5	IgG	Mouse	0.5 0.25 0.125	35 30 25	Yes	N/A	[64]
2AC7		IgG1	Mouse	0.5 0.25 0.125	80 75 70	Yes	Conformational epitope	[51]
9AD4		IgG2a	Mouse	0.5 0.25 0.125	70 60 55	Yes	**Linear epitope** beginning at Y346, basigin binding adjacent	[51]
QA5		IgG1	Mouse	0.5 0.25 0.125	60 50 45	Yes	**Linear epitope** beginning at Y194	[51]
6BF10		IgG	Mouse	0.5 0.25 0.125	30 30 30	Yes	Conformational epitope	[51]
QA1		IgG	Mouse	0.5	35	Yes	Conformational epitope, basigin blocking	[51]
R5.004		IgG1	Human	0.5 0.25 0.01	90 80 30	Yes	Conformational epitope, basigin blocking	[38]
R5.008		IgG1	Human	0.5 0.25 0.01	80 75 20	Yes	Conformational epitope, basigin blocking	[38]
R5.013		IgG1	Human	0.5 0.25 0.01	65 50 10	Yes	Conformational epitope, basigin blocking	[38]
R5.016		IgG1	Human	0.5 0.25 0.01	95 85 50	Yes	Conformational epitope, adjacent to basigin binding site	[38]
R5.017		IgG1	Human	0.5 0.25 0.01	90 80 30	Yes	Conformational epitope, basigin blocking	[38]
R5.018		IgG1	Human	0.5 0.25 0.01	50 35 5	Yes	Conformational epitope, basigin blocking	[38]
R5.019		IgG1	Human	0.5 0.25 0.01	90 80 20	Yes	Conformational epitope, basigin blocking	[38]
5G6	PfRipr	IgG	Mouse	0.5 0.25 0.125	35^b^ 25^b^ 15^b^	Unknown	Conformational epitope	[45]
1G12		IgG	Mouse	0.5 0.25 0.125	50^b^ 35^b^ 25^b^	Unknown	Conformational epitope	[45]
c02	PfCyRPA	IgG2b	Mouse	0.5 0.25 0.125	50^b^ 40^b^ 30^b^	Yes	Conformational epitope between amino acids 26–181	[62]
c04		IgG3	Mouse	0.5 0.25 0.125	50^b^ 40 30	Yes	Conformational epitope between amino acids 26–181	[62]
c06		IgGa	Mouse	0.25 0.125	50^b^ 40^b^	Yes	Conformational epitope between amino acids 26–181	[62]
c08		IgG1	Mouse	0.5 0.25 0.125	30^b^ 20^b^ 15^b^	Yes	Conformational epitope between amino acids 26–181	[62]
c09		IgG3	Mouse	0.5 0.25 0.125	50^b^ 40^b^ 30^b^	Yes	Conformational epitope between amino acids 26–181	[62]
c10		IgG1	Mouse	0.5 0.25 0.125	45^b^ 35^b^ 25^b^	Yes	Conformational epitope between amino acids 26–352	[62]
c12		IgG2a	Mouse	0.5 0.25 0.125	50^b^ 40^b^ 30^b^	Yes	Conformational epitope on blades 2–3	[62]
SB1.6		IgG	Mouse	0.5 0.25 0.125	65^b^ 50^b^ 35^b^	Unknown	Conformational epitope between amino acids 74–251	[65]
SB2.1		IgG	Mouse	0.5 0.25 0.125	50^b^ 40^b^ 30^b^	Unknown	Conformational epitope between amino acids 74–251	[65]
SB2.3		IgG	Mouse	0.5 0.25 0.125	50^b^ 40^b^ 30^b^	Unknown	Conformational epitope between amino acids 74–251	[65]
SB3.3		IgG	Mouse	0.5 0.25 0.125	40^b^ 30^b^ 20^b^	Unknown	Conformational epitope between amino acids 74–251	[65]
5B12		IgG	Mouse	2	70^b^	Unknown	Conformational epitope	[63]
3D1		IgG	Mouse	2	70^b^	Unknown	Conformational epitope	[63]
8A7		IgG1	Mouse	2	70^b^	Unknown	Conformational epitope on blades 2–3	[63]

a Abbreviation: GIA, growth inhibition activity.

b Based on only two-cycle growth inhibition while all others are based on one cycle.

Box 2 **Assay of Growth-inhibition Activity**

The growth-inhibition activity (GIA) assay is an essential tool for the development of a blood-stage malaria vaccine. The general procedure involves allowing a tightly synchronized culture of ring-stage parasites, usually achieved through sorbitol treatment, to mature into schizonts, which then rupture and reinvade red blood cells in the presence or absence of an antibody of interest. The number of viable parasites in the culture after reinvasion can be assessed through measurements of parasite lactate dehydrogenase. A % GIA is then calculated based on relative invasion of parasites when the antibody is present compared to when it is absent. Sometimes, this assay is carried out over two cycles of invasion and growth, known as a two-cycle GIA, which generally shows higher inhibition for the same antibody than a one cycle [23]. The standardized assay of GIA used by the GIA Reference Centre at the NIH uses the one-cycle methodology [80].

The GIA assay has been shown to be a useful correlate of vaccine-induced protection using MSP1-, AMA1-, and RH5-based vaccines in nonhuman primates challenged with *P. falciparum* or *P. knowlesi* [9,81,82]. However, it is not without controversy. Studies in endemic countries have had varying results regarding the usefulness of this assay in predicting natural clinical immunity. In a Kenyan cohort, increasing exposure to malaria did not correlate with higher levels of GIA when tested on the serum of exposed individuals [83]. However, in children, inhibitory antibodies seem to play a more important role [83,84]. In a separate study carried out in Mali, the level of GIA activity from the serum could not determine whether an individual was protected or susceptible to infection [85]. Recently, however, an *Aotus* study showed that the passive transfer of a GIA-positive anti-PfRH5 (clone 2AC7) antibody with no Fc effector functions is able to protect from infection [49]. This suggests that even if erythrocyte entry blocking antibodies are not a strong contributor to natural malaria immunity, they could still be induced by vaccination to confer a ‘non-natural’ form of protection. This concept is no different to vaccines targeting the sporozoite and/or sexual stages of the parasite, against which strong responses also fail to arise following natural malaria exposure.

Alt-text: Box 2

The primary mechanism through which inhibitory antibodies to PfRH5 are thought to work is by disrupting the PfRH5–basigin interaction [20,38]. This is likely how anti-PfRH5 mAbs R5.004, R5.008, R5.013, R5.017, R5.019, and QA1 (which clearly block basigin in surface plasmon resonance experiments and/or based on their crystal structures) inhibit parasite invasion (Figure 1C) [38,51]. However, 2AC7 and R5.016, the two most potent anti-PfRH5 mAbs, and 9AD4, inhibit invasion seemingly without blocking the PfRH5:basigin interaction *in vitro*, despite binding close to the basigin-binding site (Figure 1D) [38,51]. It was suggested that 2AC7 works by disrupting the PfRH5-PfCyRPA interaction [45]. However, this is inconsistent with data showing that 2AC7 competes for binding with R5.016, on the opposite side of PfRH5 from where PfCyRPA interacts [38]. Further, the few published anti-PfRH5 mAbs that block PfCyRPA binding are noninhibitory [38]. It is clear that the most potent neutralizing anti-PfRH5 antibodies all bind towards the tip of the PfRH5 kite, making this a promising candidate for **structure-guided immunogen design** to increase relative concentrations of neutralizing to non-neutralizing antibodies (Figure 1) [53].

### PfRH5-based Vaccines in Clinical Development

Among members of the RCR complex, vaccine development for PfRH5 is the most advanced [54]. A study in *Aotus nancymaae* showed that both a protein-in-adjuvant formulation, using non-human-compatible **Freund’s adjuvant**, as well as human-compatible chimpanzee adenovirus-poxvirus prime-boost vectored vaccines, effectively prevented malaria after a **heterologous challenge** [9]. The protein-in-adjuvant formulation resulted in sterile protection in three of six animals, with the other three of six animals self-curing after challenge, while never exceeding 10 000 parasites/μl [9].

In an initial Phase Ia clinical trial, PfRH5 was delivered through intramuscular injection of recombinant chimpanzee adenovirus serotype 63 (ChAd63) with or without a modified vaccinia virus Ankara (MVA) poxvirus boost 8 weeks later [42]. Both viruses are replication-defective in humans but express the full-length PfRH5 antigen *in situ* from virally infected cells. The results from this study showed a favourable safety profile and the induction of anti-PfRH5 antibodies of approximately 10 μg/ml at peak, far exceeding the levels acquired through natural malaria infection [42]. The vaccine-induced anti-PfRH5 polyclonal antibodies exhibited cross-strain functional GIA *in vitro* and inhibited key interactions within the RCR complex as well as between PfRH5 and basigin (as assessed using ELISA-based assays). A Phase Ib field trial in Tanzania of the viral vectored PfRH5 vaccine is ongoing, with a dose-escalation and age-de-escalation study design (Clinicaltrials.gov NCT03435874). Subsequently, a second-generation full-length PfRH5 protein vaccine was designed, using a *Drosophila* S2 stable cell line system for expression and **C-tag purification** technology [55., 56., 57.]. This protein, termed RH5.1, has been through a Phase I/IIa clinical trial in healthy UK adults formulated in GSK’s AS01_B_ adjuvant (Clinicaltrials.gov NCT02927145), including a blood-stage *P. falciparum* controlled human malaria infection study to assess vaccine efficacy [58]. Although reporting of these results is awaited, these data are likely to lay important foundations on which future vaccines targeting the wider RCR complex will build.

As vaccine design is often an iterative process, preclinical development of improved PfRH5-based vaccines has continued as the earlier vaccine formulations make their way through clinical evaluation. This includes the successful **heat stabilization** of PfRH5, a potentially important characteristic for a vaccine component, which also enabled inexpensive and scalable expression in *Escherichia coli* [59].

## *P. falciparum* RH5-interacting Protein (PfRipr)

### Structure and Function

PfRipr is an approximately 120 kDa protein, first identified when **gel filtration chromatography** of what was presumed to be **ion-exchange chromatography** purified PfRH5 showed a 150–200 kDa compound, instead of the expected 45 kDa size of the processed fragment [18]. Mass spectrometry revealed the presence of another protein, complexed with PfRH5, corresponding to hypothetical protein, PFC1045c, later renamed PfRipr [18]. This interaction was confirmed by immunoprecipitation [18]. Independently, PFC1045c was identified as a member of the *P. falciparum* ‘invadome’, a collection of proteins that are hypothesized to be involved in invasion [60].

PfRipr is composed of putatively unstructured regions, 10 epidermal growth factor-like (EGF) domains and has 87 cysteines. The full-length 120 kDa protein is processed into two fragments of similar size, an N-terminal fragment (including EGF domains 1 and 2) and a C-terminal fragment (including EGF domains 3–10) [18]. Whether cleavage is critical to the function of PfRipr is not clear. Like PfRH5, recombinant expression of this large and complex antigen, in its entirety, proved difficult; however, the first reported success to provide reasonable quantities was again using the *Drosophila* S2 stable cell line platform. This led to a recent 7 Å resolution cryoelectron microscopy (cryo-EM) structure of the RCR complex, revealing that PfRipr interacts with PfCyRPA through blade six of the PfCyRPA β-propeller [37]. However, the moderate resolution of this structure precluded building of an atomic model of PfRipr, and indeed, the electron density attributed to PfRipr is sufficient to include only around half of the molecule. There is still significant work to do to understand the structure of PfRipr and how it contributes to invasion.

Interestingly, PfRH5 and PfRipr localize to different compartments during the schizont stage of the *P. falciparum* life cycle: PfRipr in the **micronemes** and PfRH5 in the rhoptries [23]. Despite this, parasites with conditional knockouts of PfRipr have a similar invasion phenotype to parasites that try to invade in the presence of anti-PfRH5 antibodies; these parasites induce membrane deformation, but cannot complete invasion [24].

### Antibody Responses to PfRipr

Much like PfRH5, PfRipr is highly conserved, essential for invasion, capable of inducing cross-strain neutralizing antibodies in assays of GIA, but is not a target of naturally acquired immunity [43,46]. There are only six non-synonymous SNPs with a minor allele frequency above 5% in *RIPR* (Table 1). In line with this, polyclonal antibodies raised against EGF domains 6–8 (amino acids 791–900) of PfRipr are cross-strain neutralizing [18,45]. In one report, GIA assays using the FCR3 laboratory-adapted strain and the 3D7 clone of *P. falciparum* showed nearly complete versus 50% growth inhibition at 3 mg/ml total rabbit IgG, respectively [18]. In a separate study, polyclonal anti-PfRipr total rabbit IgG was produced using a fragment of PfRipr spanning amino acids 279–995 and then tested for GIA with homologous (3D7 clone) and heterologous (FVO strain) parasites, both of which were successfully inhibited by the purified polyclonal IgG [61].

Against the entirety of the large PfRipr molecule, only two neutralizing mAbs have been mapped so far, targeting EGF-7 [45]. The most potent mAb, 1G12, has an EC_50_ of 0.35 mg/ml, approximately tenfold less potent than the best PfRH5 mAbs (Table 2) [45]. Notably, the only region of PfRipr known so far to be targeted by neutralizing mAbs (EGF-7) appears to be located away from the PfCyRPA:PfRipr interface, which is towards the N-terminal half of the protein. Accordingly, the mechanism behind neutralization for anti-PfRipr antibodies does not appear to be related to RCR complex formation and remains to be elucidated [45].

## *P. falciparum* Cysteine-rich Protective Antigen PfCyRPA

### Structure and Function

PfCyRPA is a 43 kDa protein that was identified as a blood-stage vaccine candidate antigen through reverse vaccinology based on a predicted N-terminal secretion peptide, upregulation during the blood stage of infection, and a putative role in erythrocyte invasion owing to proximity to other invasion genes such as *PfRH5* [62]. Transcriptional analyses also identified PFD1130w as part of the *P. falciparum* invasion pathway [60].

Subsequently, and notably after it had already been independently identified as a vaccine candidate, PfCyRPA was coimmunoprecipitated with PfRH5 and PfRipr [23,41,62]. Akin to PfRH5 and PfRipr, PfCyRPA is highly conserved, with only a single SNP above 5% prevalence (Table 1), essential for invasion as conditional knockdown causes the loss of invasion activity [24], and has poor sero-reactivity from natural exposure [47,62]. PfCyRPA localizes to the parasite micronemes, as is the case with PfRipr, but different from PfRH5 [23,24]. Two crystal structures of PfCyRPA have been solved, revealing a six-bladed β-propeller with five disulfide bonds (four intra-sheet, one inter-sheet) [63,64]. CyRPA has some similarity to known sialidases, but it lacks critical catalytic residues [63]. Further ELISA-based analyses, now confirmed by the cryo-EM structure, show that PfCyRPA acts as a bridging protein, which links PfRH5 and PfRipr [25,37]. No further role of the protein beyond this has been reported.

### Antibody Responses

Like PfRH5 and PfRipr, PfCyRPA is a weakly immunogenic target during natural infection [47,62]. However, monoclonal antibody binding analyses have shown that PfCyRPA is also susceptible to cross-strain neutralizing antibodies [62]. Dreyer *et al.* showed that, of their panel of nine mAbs (derived from mice immunized with full-length soluble PfCyRPA protein produced in mammalian HEK293 cells), seven were cross-strain inhibitory, with the best achieving GIA of 58% at 1 mg/ml against 3D7 clone parasites (Table 1). All seven of the inhibitory antibodies bound a **conformational epitope** between amino acids 26 and 181 [62].

**Figure 2:**
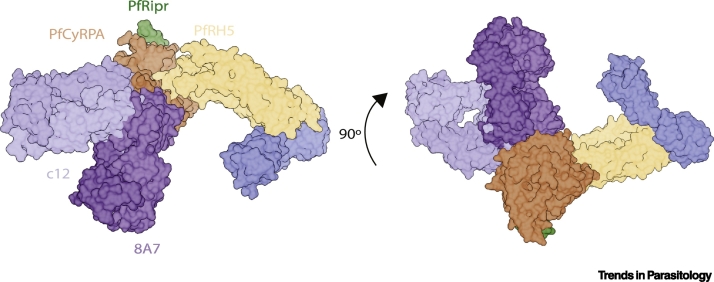
Mapping the Critical PfCyRPA Epitopes on the RCR Complex Bound to Basigin. PDB IDs: RCR complex (6MPV), PfRH5:basigin (PDB: 4U0Q), PfCyRPA:c12 Fab (PDB: 5EZO), and PfCyRPA:8A7 Fab (PDB: 5TIH). Figure produced using Chimera [87]. Abbreviations: RCR, PfRH5-PfCyRPA-PfRipr; PDB, protein data bank.

Analysis of a different panel of five mouse-derived antibodies identified three inhibitory anti-PfCyRPA mAbs [63]. The mechanism for the most potent of these, 8A7, was reported to be disruption of the PfCyRPA-PfRH5 interface [45,63]. This is in contrast to data on eight anti-PfRH5 mAbs, spanning three epitope regions, which all disrupted the same PfCyRPA-PfRH5 interaction *in vitro* and yet had negligible GIA against *P. falciparum* [38]. Reconciling these observations will require further study. Interestingly, two additional anti-PfCyRPA mAb clones from the same study, 5B12 and 3D1, competed for binding with 8A7 on PfCyRPA and yet did not disrupt RCR complex formation *in vitro*; instead they stably formed a quaternary complex [45]. The crystal structure of 8A7 Fab bound to PfCyRPA has also been solved [63]. It is not immediately clear how it would block the PfRH5:PfCyRPA interface, as superimposition of 8A7:PfCyRPA onto the PfRH5:PfCyRPA cryo-EM structure does not indicate that they would compete for binding (Figure 2).

These data would thus suggest that there is another mechanism of action, beyond disruption of RCR complex formation, which allows for anti-PfCyRPA antibodies to block parasite invasion. The authors of one study noted that inhibitory anti-PfCyRPA antibodies that bind the RCR complex point towards the erythrocyte membrane [45]. From there, it was speculated that there could be steric interference between the bulky antibody Fc constant domain and the membrane, preventing the RCR complex from binding to erythrocyte basigin [37].

Monoclonal antibodies targeting PfCyRPA have also been tested in animal challenge models showing some efficacy. In a **NOD-*scid* IL2Ry**^**null**^ mouse (NSG) model grafted with human erythrocytes, there was a marked decrease (90%) in parasitaemia after *P. falciparum* challenge when pretreated with 2.5 mg of monoclonal antibody SB1.6 or C12 (Table 2) [65]. A follow-up study showed that rabbit immunization with PfCyRPA integrated into influenza virosomes elicits neutralizing antibodies that slow parasite growth in the NSG mouse model [66].

At the time of writing, there have been no human vaccine trials using PfRipr- or PfCyRPA-based immunogens. We can expect that candidate vaccines will begin to appear in animal studies and then human trials in the coming years given the strong scientific rationale for exploring such vaccines and the promising preclinical studies so far.

## Targeting the PfRH5-PfCyRPA-PfRipr Complex

Owing to the different subcellular locations of PfRH5 versus PfCyRPA and PfRipr, the RCR complex likely only forms during invasion when the components are secreted from either the rhoptries or micronemes, at which point the complex forms on the surface of the invading merozoite [23,24]. It was also recently shown in the rhesus malaria *Plasmodium knowlesi*, that PkRipr forms a complex with two other proteins: cysteine-rich small secreted protein (PkCSS) and thrombospondin-related apical membrane protein (PkPTRAMP) [28]. *P. knowlesi* lacks an orthologue of PfRH5 altogether; and, despite being present, PkCyRPA does not, surprisingly, interact directly with PkRipr, although both are still essential for invasion [28]. This then raises the question of whether the potentially different ancestral roles of Ripr and CyRPA are retained in *P. falciparum* alongside their established function within the RCR complex mediating the PfRH5–basigin interaction. Moreover, it is not currently known what the functions of PfCSS or PfPTRAMP are in *P. falciparum*, and whether they still interact with PfRipr. Encouragingly, however, for vaccine development against the other human malaria parasite species, the authors also reported strong GIA when using antisera raised against the C-terminal EGF domain region of PkRipr [28].

A critical question for future vaccine development is the merits of targeting the wider set of RCR complex antigens, and to what extent this could lead to much higher levels of vaccine efficacy than targeting a single component alone. Indeed, spreading immune pressure over at least three essential antigens, rather than just one, could be viewed as a distinct advantage. Studies are in their early stages; however, there has been some work examining the growth-inhibitory activity of mixtures of antibodies that target the three RCR complex components. Evidence suggests that antibody mixtures to PfCyRPA, PfRH5, and/or PfRipr typically act additively, and in some cases synergistically, when combined in both *in vitro* and *in vivo* experiments [23,45,64,67,68]. These differences potentially relate to the antibody clones that may dominate different polyclonal responses following immunization of different species with different vaccine formulations. In any case, either scenario (additivity and/or synergy) should be highly beneficial to vaccine efficacy outcome, assuming that a mixture of vaccine antigens maintains the same quantitative levels of immunogenicity and does not lead to significant immune interference.

Recently, it was found that specific noninhibitory anti-PfRH5 mAbs are able to synergize with inhibitory anti-PfRH5, -PfCyRPA, and -PfRipr mAbs by slowing down parasite invasion [38]. These dilatory antibodies are presumed to enhance the activity by giving the inhibitory mAbs more time to bind during parasite invasion (Figure 1E) [38]. This also points to potentially underappreciated roles for 'non-neutralizing epitopes' in structure-guided vaccine design, whereby non-neutralizing epitopes are routinely presumed to have little to no role in conferring protection.

Another area of exploration that beckons is the possibility of developing a vaccine using the assembled PfRH5-PfCyRPA-PfRipr complex – one proof-of-concept for this being that vaccination with a different merozoite multiprotein invasion complex, PfAMA1-PfRON2, was more protective than either protein on its own [69]. Another is that, to date, all reported functional mAb clones against PfRH5 and PfRipr, and three/four clones against PfCyRPA, bind epitopes that are exposed on the RCR complex surface [38,45]. However, the first results using this approach have not been particularly promising. Here, polyclonal IgG from mice vaccinated with the assembled RCR complex was no more potent in GIA assays than IgG from mice immunized with PfRipr, PfCyRPA, or PfRH5 on their own [45]. However, it remains unclear whether the trimeric complex was stable in the adjuvant formulation used, and also how the observed immune interference impacted overall antibody potency [45]. It will be important to deconvolute these issues in future studies and also assess the merits of vaccinating with the trimeric complex as opposed to one, two, or three separate immunogens.

## Concluding Remarks

The antigens comprising the PfRH5-PfCyRPA-PfRipr complex are promising vaccine targets given their ability to induce strain-transcending neutralizing antibodies against blood-stage *P. falciparum*. This is due to their high degree of sequence conservation as well as the lack of redundant pathways that can substitute the function of the complex. However, many questions about the biological role of this complex remain unanswered, including the mechanism of invasion-inhibition for anti-PfCyRPA and anti-Ripr antibodies where inhibitory antibodies do not necessarily block complex formation (see Outstanding Questions).

Nevertheless, vaccines targeting this complex still face challenges, most notably the need for high concentrations of neutralizing antibody to block erythrocyte invasion. The required concentration to achieve parasite neutralization for even the most potent antibodies is orders of magnitude higher than for other bacterial or viral pathogens [70., 71., 72.]. Encouragingly, anti-PfRH5 can neutralize parasite invasion with tenfold less antigen-specific IgG than the previously leading vaccine candidates PfAMA1 and PfMSP1 [8]. Further optimization of the vaccine antigen, or combining multiple antigens, could potentially reduce these concentrations even further (see Outstanding Questions).

Insights gleaned from structure-based vaccinology are contributing to a growing understanding of critical inhibitory epitopes around the RCR complex. Continued efforts to understand and map the most antibody-susceptible epitopes should enable the development of a highly immunofocussed vaccine, designed to neutralize specific and vulnerable regions of the complex. Indeed, such ‘precision vaccinology’ approaches are already bearing fruit in clinical trials of novel vaccines targeting viral pathogens (see Outstanding Questions) [73].

Moving forward with human clinical trials for the most promising vaccine formulations will be crucial to determine the feasibility of a PfRH5-PfCyRPA-PfRipr-based vaccine, especially given the natural limitation of the *in vitro* GIA assay to offer a readout over the whole immune system. To date, progress moving from initial vaccine target identification (in 2011) to the first-in-human clinical trial (initiated in 2014) has been swift, as exemplified by PfRH5 [39,42]. The same will need to be done with PfCyRPA and PfRipr in order to establish whether vaccines targeting the PfRH5-PfCyRPA-PfRipr complex can become part of the global malaria toolkit.

Outstanding Questions How do anti-PfCyRPA and anti-PfRipr antibodies block erythrocyte invasion?

What are the roles of PfCyRPA and PfRipr in erythrocyte invasion?

Will an engineered immunogen with only the critical basigin binding region of PfRH5 be more effective at focusing the immune response?

Are the antibody concentrations induced through conventional vaccination approaches in humans compatible with anti-RCR vaccine-mediated protection?

How well do assays of growth inhibition activity correlate with human *in vivo* efficacy against blood-stage malaria?

Can an RCR-based vaccine induce protective immunity in humans?

Alt-text: Outstanding Questions

## Glossary

**Blood-stage vaccine**: a vaccine that targets the asexual parasite while it replicates in the blood, in contrast to pre-erythrocytic vaccines or sexual-stage transmission-blocking vaccines.
**C-tag purification**: a method of affinity purification that recognizes a four amino acid ‘tag’, EPEA, at the C terminus of a protein.
**Conformational epitope**: an antibody-binding site only recognized in its secondary or tertiary structure, resulting in the loss of binding upon denaturation.
**EC**_**50**_: the concentration of a drug required to achieve 50% of the maximum effect.
**Freund’s adjuvant**: an oil-in-water emulsion delivered alongside a vaccine antigen to stimulate the immune system (used in animal models but not humans). Incomplete Freund’s adjuvant (used for booster immunizations) lacks the mycobacterial component present in complete Freund’s adjuvant (used for the initial priming immunization).
**Gel filtration chromatography**: the separation by chromatography of molecules based on size.
**Glycosylphosphatidylinositol (GPI) anchor**: a post-translational lipid anchor commonly used among eukaryotic cells for attaching cell-surface proteins to the cell membrane.
**Growth-inhibition activity (GIA) assay**: a method used for determining parasite growth during the blood stage of infection and the impact of drugs or antibodies on parasite growth.
**Heat stabilization**: increasing the tolerance of a protein for elevated temperatures without denaturation.
**Heterologous challenge**: deliberate infection with a *P. falciparum* strain that is different from the one incorporated in the vaccine.
**Ion-exchange chromatography**: separation by chromatography of molecules based on their ionic interactions.
**Linear epitope**: an antibody binding site that is recognized based on its primary structure (ie. the linear sequence of amino acids), and thus can still be recognized after protein denaturation
**Microneme**: a specialized organelle of apicomplexan parasites primarily involved in the secretion of protein, similar to the rhoptries.
**Monoclonal antibodies (mAbs)**: antibodies that are derived from a single parent B cell and are thus all identical.
**NOD-*scid* IL2Rγ**^**null**^**mice**: a type of immunodeficient mice lacking both T and B cells.
**Parasitophorous vacuole**: a membrane-bound organelle of apicomplexan parasites in which the organism develops.
**Passive transfer**: the delivery of antibodies directly, as opposed to induction of antibody expression through immunization.
**Pre-erythrocytic vaccine**: a vaccine that targets the parasite before it enters the blood from the liver.
**Rhoptries**: specialized organelles of apicomplexan parasites primarily involved in the secretion of protein, similar to the micronemes.
**Structure-guided immunogen design**: the process of using structural information about an antigen to create a synthetic antigen with specific desired properties.
